# Use of adrenaline continuous infusion to treat hypotension during general anaesthesia in a cow and a calf

**DOI:** 10.1186/s13620-020-00164-0

**Published:** 2020-07-02

**Authors:** Laura Gómez Fernández, María Chie Niimura del Barrio, Claire Loughran

**Affiliations:** 1 UCD Veterinary Hospital, University College Dublin, Belfield, Dublin 4, Eircode: D04 V1W8 Ireland; 2 Lumbry Park Veterinary Specialists, Alton, Hampshire United Kingdom

**Keywords:** Anaesthesia, Bovine, Adrenaline, Epinephrine, Infusion

## Abstract

**Background:**

Hypotension is one of the most common complications observed during inhalation anaesthesia in veterinary patients. Treatment of hypotension in cattle is more challenging than in other species, owing to the limited number of drugs licensed in food producing animals. The use of adrenaline as an infusion to support blood pressure has not been described previously in bovines.

**Case presentation:**

A cow and a calf presented to University College Dublin Veterinary Hospital for bilateral mandibular fracture repair and bladder rupture repair respectively, developed severe anaesthetic related hypotension unresponsive to conventional treatments. In both cases an adrenaline infusion was started and slowly increased to effect, with infusion rates ranging from 0.01 to 0.25 μg/kg/min. Blood pressure increased as the adrenaline infusion rate increased, but clinically significant improvements in blood pressure were only observed with infusion rates exceeding 0.05 μg/kg/min. The side effect observed with adrenaline infusion was an increase in plasma lactate levels in both cases. Both animals were euthanised due to non-anaesthetic related complications.

**Conclusions:**

Maintenance of normotension is important during bovine anaesthesia to prevent the development of post-anaesthetic complications. In the cases described here, adrenaline was effective as an additional treatment of anaesthetic related hypotension. Further research is required to establish the recommended infusion rates, cardiovascular effects and possible side effects of adrenaline infusion administration as a treatment for hypotension in bovines.

## Background

Hypotension is one of the most common complications observed during inhalation anaesthesia in veterinary [1] and human patients [2].

There is a lack of consistency in the definition of hypotension within the human and veterinary literature despite extensive research in this area [3, 4]. In veterinary medicine, a survey published in 2015 suggested a definition of hypotension as a mean arterial pressure (MAP) below 62 mmHg in anaesthetised dogs [4], while hypotension in horses was defined as MAP < 70 mmHg [5]. This higher MAP in horses was related to a well-recognised link between post-anaesthetic myopathy, and hypotension during anaesthesia [5–8]. Two studies in mechanically ventilated halothane anaesthetised horses, demonstrated that 8% of horses whose MAP was maintained between 80 and 90 mmHg developed transient post-anaesthetic myopathy. In contrast, 100% of horses whose MAP was maintained between 50 and 65 mmHg developed post-anaesthetic myopathy, with 38% of horses requiring euthanasia [9, 10]. This link between hypotension and post-anaesthetic myopathy has also been shown clinically. Young and Taylor [11] demonstrated that 0.9% of horses with untreated hypotension (MAP < 70 mmHg) during anaesthesia developed fatal post-anaesthetic myopathy, compared to 0% of horses that had hypotension during anaesthesia treated with fluids and dobutamine. Despite the extensive evidence supporting the definition of hypotension in horses, there is no clear definition of hypotension in bovines. However, as bovines have a similar shape and body mass when compared to horses, for the purposes of this case report the definition of hypotension will be extrapolated from that of horses in that MAP < 70 mmHg.

The frequency of hypotension in dogs during general anaesthesia has been reported to range from 7 to 37.9%, and similar levels of hypotension (8.5–33%) have been reported in cats [1, 12, 13]. However, the frequency of anaesthetic-induced hypotension in large animals has not been reported.

Anaesthetic-related hypotension can be attributed to 1) arterial vasodilation, 2) low cardiac preload, 3) bradycardia or 4) decrease in myocardial contractility [14–16]. There are several treatment options used to increase blood pressure (BP) in hypotensive animals, these include: decreased anaesthetic-induced vasodilation by decreasing delivery of inhaled anaesthetic; heart rate assessment and treatment of bradycardia with anticholinergics; increased intravenous fluid administration to improve venous return and cardiac output; administration of inotropic drugs to improve myocardial contractility and administration of vasopressors, if vasodilation is suspected (e.g. sepsis) [5, 15, 17, 18]. Other methods to improve BP include: decreased peak inspiratory pressure if positive pressure ventilation is being performed, use of locoregional techniques [15], use of minimum alveolar concentration (MAC) sparing drugs (e.g. ketamine, ⍺-2 adrenergic agonists) [5], and administration of calcium gluconate intravenously (IV) to improve cardiac contractility [19].

Bovines are food producing animals, hence, limited number of drugs are licensed for use in these animals. As a result, the treatment of hypotension is more challenging than in non-food producing species. In Ireland, the European Commission (EU) N° 37/2010 regulates the drugs that can be administered for the treatment of hypotension in food producing animals; these include intravenous crystalloid fluids (isotonic and hypertonic), atropine, adrenaline, ⍺-2 adrenergic agonists (xylazine and detomidine), and ketamine. Adrenaline is the only positive inotrope drug licenced in food producing animals. Adrenaline is an endogenous catecholamine that acts at ⍺-1 receptors producing an increase in systemic vascular resistance and, β-1 receptors producing an increase in heart rate and contractility. Aprea et al. [20] described the successful use of adrenaline as an intravenous (IV) bolus (30 μg/kg) to resuscitate a calf after cardiac arrest. The use of adrenaline as a continuous infusion to support BP has not been described previously. This case report describes the use of adrenaline administered as a continuous infusion to treat hypotension during general anaesthesia in a cow and a calf.

## Case presentation

### Case 1

A 2 year-old female Friesian Holstein weighing 480 kg, was presented to University College Dublin Veterinary Hospital (UCDVH) for bilateral mandibular fracture repair which occurred the previous day. The cow had calved 1 month prior to presentation.

The cow presented depressed with 8–10% dehydration (eyes markedly sunken, 4 seconds (s) of skin tent) and 3 s capillary refill time (CRT). On auscultation, lung and heart sounds were normal. Heart rate (HR) was 90 beats per minute (bpm), respiratory rate (RR) was 33 breaths per minute and rectal temperature was 39 °C. On admission packed cell volume was 28% and total protein was 71 g/L. The cow presented with bilateral mandibular swelling. The cow was sedated with 12 μg/kg detomidine (Medesedan; Chanelle Pharma, Loughrea, Galway, Ireland) IV to allow intra-oral examination and radiography. Radiographs revealed a bilateral complete mandibular fracture with associated secondary soft tissue swelling.

The cow was medically stabilised prior to fracture repair, which was planned for the following day. Treatment included the administration of 20 L of water supplemented with 500 g of sodium bicarbonate (Shamrock bread soda; Ireland), 100 g of potassium chloride (KCl) (Potassium chloride SigmaUltra; Sigma-Aldrich, Ireland), 300 mL of propylene glycol (Chanatol oral solution; Chanelle Pharma, Loughrea, Galway, Ireland) and a ruminant feed supplement product (StimuVet; Duggan Veterinary Supplies Ltd., Thurles, Tipperary, Ireland) delivered via nasogastric tube, 1 mL/25 kg of penicillin G and streptomycin (Pen & Strep suspension for injection; Norbrook, Newry, Nothern Ireland, UK) intramuscularly (IM) and 2.2 mg/kg of flunixin meglumine (Flunixin injection; Norbrook, Newry, Nothern Ireland, UK) IV.

A further 10 L of water supplemented with 500 g of sodium bicarbonate and 100 g of KCl was administered every 4 h overnight via nasogastric tube. The bicarbonate used in this case was non-pharmaceutical grade, but was made to a high standard for oral consumption by humans and was deemed of acceptable quality to be used for oral administration in bovines. It also presented the only economically viable option for oral bicarbonate administration.

Clinical examination on the day of the surgery revealed approximately 6% dehydration, normal mentation, HR of 60 bpm, RR of 16 breaths per minute and rectal temperature of 38.6 °C. A pre-anaesthetic venous blood gas and electrolytes were analysed (Table 1). All parameters were within normal limits, except for calcium, which was low (1.14 mmol/L).

**Table 1:** Details of the pre and intra-operative venous and arterial blood gas analysis of an adult cow with bilateral mandibular fracture. Reference intervals were taken from equine and bovine references [21–23]

Variables	Reference interval	Admission venous blood gas	Intra-operative arterial blood gas
**pH**	7.42–7.47	7.45	7.46
**PaO**_**2**_**(kPa)**	13.33–66.67	8.35	29.32
**PaCO**_**2**_**(kPa)**	4.89–6.17	6.78	5.68
**Bicarbonate (mmol/L)**	26.7–30.30	25.1	29.3
**Base excess (mmol/L)**	- 0.51 to + 8.80	+ 1.2	+ 4.9
**Lactate (mmol/L)**	0.42–0.75	0.43	1.2
**Sodium (mmol/L)**	133–141	140.5	132.4
**Potassium (mmol/L)**	3.05–4.65	4.07	4.09
**Ionised calcium (mmol/L)**	1.34–1.72	1.14	1.10
**Chloride (mmol/L)**	100–110	108	102

Partial pressure of oxygen in arterial blood (PaO_2_), partial pressure of carbon dioxide in arterial blood (PaCO_2_)

A 14-gauge catheter-over-the needle (MILA International, INC; UK) was placed in the left jugular. Prior to premedication the cow received 5 L of Hartmann’s solution (Lactated Ringer’s Solution, Vetivex® 11; B Braun, Germany) IV. The cow was premedicated with 0.1 mg/kg xylazine (Chanazine 2%; Chanelle Pharma, Loughrea, Galway, Ireland) and 0.1 mg/kg butorphanol (Butador®; Chanelle Pharma, Loughrea, Galway, Ireland) IV which resulted in adequate sedation. General anaesthesia was induced with 3 mg/kg ketamine (Ketamidor®, Chanelle Pharma, Loughrea, Galway, Ireland) IV. The cow remained in a sternal position immediately after induction. The upper jaw was abducted with a tie and manual intubation of the trachea was performed with a 26 mm internal diameter (ID) cuffed endotracheal tube (Jorgensen Labs, UK). The cow received an additional 0.5 mg/kg ketamine IV before being hoisted into theatre. In theatre the cow was positioned in dorsal recumbency, connected to a large animal circle breathing system (Model 2800C-P large and small animal anaesthesia ventilator system; Mallard Medical Inc., UK) and anaesthesia was maintained with isoflurane (Vetflurane; Virbac Animal Health, UK) delivered in up to 80% oxygen. Throughout the procedure end-tidal isoflurane (ETiso) ranged from 0.91 to 1.7%. The lungs were mechanically ventilated to maintain an end-tidal carbon dioxide (ETCO_2_) of 4–5.4 kPa with a tidal volume (TV) of 11–15 mL/kg and a peak inspiratory pressure (PIP) of 20–26 cmH_2_O. Monitoring was attached and a 22-gauge catheter (Optiva® 2; Smiths Medical International Ltd., UK) was aseptically placed in the left auricular artery. Monitoring during anaesthesia included HR, RR, temperature, ETCO_2_, ETiso, oxygen saturation (SpO_2_), electrocardiogram (ECG), IBP using a multiparameter monitor (B40 Patient monitor; GE Healthcare, Ireland), and arterial blood gas monitoring (RAPIDpoint® 500 System; Siemens Healthineers, Ireland). An arterial sample was collected anaerobically and processed within 15 min of collection. Active warming was provided throughout anaesthesia with a forced air warming blanket (3 M™ Bair hugger™ System). The cow received 5–10 mL/kg/hr. of Hartmann’s solution during anaesthesia and was regularly assessed for ruminal bloating.

Prior to surgery, a right mandibular nerve block was performed with 0.8 mg/kg lidocaine 2% (Lidocaine hydrochloride 2%; B. Braun Medical Inc., EU), using a blind technique and a 9 cm spinal needle (Spinocan® 18G 90 mm; B. Braun Medical Limited, Dublin, Ireland). Additional analgesia was provided with 0.1 mg/kg butorphanol IV given every 90 min and a ketamine infusion (10 μg/kg/min).

Thirty-five minutes after induction of anaesthesia marked hypotension (MAP 44 mmHg) was detected. Heart rate at this time was 40–45 bpm and ETiso was 1.2%. Depth of anaesthesia was reduced by decreasing ETiso to 1.0% and, Hartmann’s solution rate was increased from 5 mL/kg/h to 10 mL/kg/h. Bradycardia was treated with 5 μg/kg of atropine (Atropine sulphate; Mercury Pharma, Dublin, Ireland) IV, twice within 10 min. However this had minimal effect on the MAP. The ketamine infusion was increased to 20 μg/kg/min and ETiso was further reduced to 0.9%, MAP increased to 49 mmHg. Despite the steps taken, BP and HR failed to improve significantly during the initial 60 min and the cow remained both hypotensive and bradycardic. An adrenaline (Adrenaline (epinephrine); Mercury Pharma, Dublin, Ireland) infusion was then started, with an initial infusion rate of 0.017 μg/kg/min and gradually increased to 0.05 μg/kg/min over 10 min. During the first 20 min of the infusion, MAP gradually increased to 65 mmHg. Despite no further increases in the rate of adrenaline infusion, MAP continued to increase to 82 mmHg. The adrenaline infusion was stopped 130 min after induction of anaesthesia and ETiso increased from 1.0 to 1.3%. Heart rate remained stable (40–45 bpm) and no arrhythmias were noted during the adrenaline infusion.

A right and left mandibular blocks were performed with 0.4 mg/kg and 0.8 mg/kg of lidocaine 2% respectively 155 min after the onset of anaesthesia. A transient decrease in MAP to 70–75 mmHg was observed after the bilateral mandibular blocks, but MAP gradually increased as surgery continued. End-tidal isoflurane was increased from 1.3 to 1.7% to maintain an adequate depth of anaesthesia.

An arterial blood gas sample (Table 1) was analysed 30 min after the onset of the adrenaline infusion. Calcium remained low (1.1 mmol/L) and lactate concentration had increased from 0.43 to 1.2 mmol/L.

Both mandibular fractures were internally reduced and fixated with screws, starting with the right mandible. The ketamine infusion was stopped 20 min before the end of anaesthesia. The total duration of anaesthesia and surgery was 230 and 180 min respectively. At the end of anaesthesia the cow was hoisted into a padded recovery box, and placed in sternal recumbency where the head was supported with a foam pad. Flunixin meglumine (2.2 mg/kg) was administered IV when the cow was moved into recovery and, flow-by oxygen (15 L/min) was orotracheally delivered throughout the recovery period. The cow was extubated 23 min after the end of anaesthesia and, standing 8 min after extubation. Once standing, the cow moved freely in the recovery box with no evidence of post-anaesthetic myopathy, but the cow’s tongue was prolapsed. Four hours after recovery the cow was able to prehend food and completely retract the tongue into the mouth.

Post-operatively, the cow received analgesia, 2.2 mg/kg of flunixin meglumine IV once daily (SID) for 3 days. Antiobiotic cover consisted of, 1 mL/25 kg of penicillin G and streptomycin IM SID for 1 day, followed by 1 mL/20 kg amoxicillin and clavulanic acid (Noroclav, Norbrook Laboratories Limited; Nothern Ireland) IM twice a day (BID) for 3 days reduced to 1 mL/20 kg IM SID for a further 6 days. The cow was discharged form UCD Veterinary Hospital 8 days after surgery.

The cow was euthanised 1 month after the surgery due to wound break down.

### Case 2

An 8 week-old male Limousin calf weighing 150 kg presented to UCDVH with a history of trauma sustained the previous day. The calf had become inappetence and recumbent.

During the physical examination the calf was, dull and unable to raise his head. Heart rate was 160 bpm, RR 64 breaths per minute and temperature 38.4 °C. The abdomen was slightly distended and abdominal palpation elicited pain. An abdominal ultrasound revealed free fluid within the abdomen and a partially filled bladder. The day following admission an abdominal drain was placed and urine drained from the abdomen. A venous blood gas and blood glucose were analyzed and revealed hyperkalaemia (6.31 mmol/L) and hypoglycaemia (2.6 mmol/L) respectively. An intravenous 18-gauge catheter was placed in the left jugular. The calf received Hartmann’s solution supplemented with 2.5% glucose (Glucose 50% w/v sterile concentrate, B Braun Medical Ltd., Dublin, Ireland) at 10 mL/kg/hr. A second venous blood gas and blood glucose were performed 30 min after the first and potassium values were normal with mild hyperglycaemia (Table 2).

**Table 2:** Details of the pre-operative venous blood gas analysis of an 8 week-old calf with bladder rupture. Reference intervals were taken from Dillane et al. [24]

Variables	Reference values	Admission venous blood gas	Pre-operative venous blood gas
**pH**	7.37–7.47	7.41	7.35
**PvCO**_**2**_**(kPa)**	5.77–7.82	6.54	7.01
**Bicarbonate (mmol/L)**	28.0–36.9	30.4	28.6
**Base excess (mmol/L)**	+ 2.6 to + 10.8	+ 5.7	+ 2.6
**Lactate (mmol/L)**	0.42–0.75	3.22	2.16
**Sodium (mmol/L)**	133.3–140.2	134.9	132.4
**Potassium (mmol/L)**	4.13–5.41	6.31	4.85
**Ionized calcium (mmol/L)**	1.17–1.37	1.02	1.11
**Chloride (mmol/L)**	93–101	96	95
**Glucose (mmol/L)**	3.9–8.4	2.6	8.7

Partial pressure of carbon dioxide in venous blood (PvCO_2_)

The calf underwent surgery for repair of a suspected bladder wall tear. Prior to premedication, a sacrococcygeal epidural with 0.1 mg/kg xylazine and 2 mg/kg lidocaine was performed. The calf was premedicated with 0.2 mg/kg butorphanol IV which resulted in adequate sedation. Anaesthesia was induced with 3 mg/kg ketamine IV. The calf remained in a sternal position immediately after induction. The upper and lower jaws were abducted using ties and the trachea was intubated with an 11 mm ID (FN11, Smiths Medical ASD, USA) cuffed endotracheal tube. The calf was hoisted into theatre and was positioned in dorsal recumbency. The calf was connected to a large animal circle breathing system and anaesthesia was maintained with isoflurane delivered in up to 80% oxygen. End-tidal isoflurane ranged from 0.3 to 1.1%. The lungs were mechanically ventilated throughout the procedure to maintain an ETCO_2_ of 4.5–5.5 kPa with a TV of 8–10 mL/kg and PIP 20–25 cmH_2_O. A 20-gauge catheter was aseptically placed in the left radial artery, after unsuccessful attempts to place a catheter in both auricular arteries. Anaesthesia was monitored as in the first case. Arterial and blood glucose samples were collected every hour. The calf received Hartmann’s solution at 10 mL/kg/hr. and a ketamine infusion was started at a rate of 10 μg/kg/min. Active warming was provided throughout anaesthesia.

Invasive blood pressure revealed marked hypotension (MAP 30 mmHg) with a HR of 80 bpm and ETiso 0.8%. End tidal isoflurane was decreased to 0.5% and, a 20 mL/kg Hartmann’s solution bolus was administered over 30 min. Mean arterial pressure increased to 35 mmHg but HR decreased to 55 bpm. The ketamine infusion was increased to 20 μg/kg/min, and an adrenaline infusion was started. The adrenaline infusion was started at 0.05 μg/kg/min and gradually increased to 0.1 μg/kg/min over a period of 25 min, during which time MAP gradually increased to 65 mmHg. Twenty five minutes after the start of the adrenaline infusion HR transiently increased from 55 to 65 bpm, but after 5 min, HR decreased to 55 bpm. Two atropine boluses of 10 μg/kg were administered IV 10 min apart, but HR did not perceptibly change. Further increases in the adrenaline infusion rate to 0.25 μg/kg/min, increased MAP to 85 mmHg by 60 min post-onset of the adrenaline infusion. The adrenaline infusion was then decreased and maintained at 0.2 μg/kg/min until the end of anaesthesia with an ETiso ranging between 0.5 to 0.9% and a MAP between 60 to 72 mmHg. No arrhythmias were noted prior to, during or after the adrenaline infusion.

Regurgitation was noticed intra-operatively and an orogastric tube was placed and left throughout the surgery and removed prior to recovery.

Table 3 shows the arterial blood gas analysis performed during the procedure. An increase in lactate concentration from 2.16 to 8.78 mmol/L was noticed during the administration of the adrenaline infusion. The first arterial blood gas analysis performed under general anaesthesia revealed hyperkalaemia (6.90 mmol/L) and hypoglycaemia (3.5 mmol/L). A bolus of 0.7 mL/kg of 50% glucose diluted in saline solution 1:2 was administered IV over 10 min. The following arterial blood analysis showed a gradual decrease on potassium levels to 4.63 mmol/L and normoglycaemia (5.6 mmol/L).

**Table 3:** Details of the intra-operative arterial blood gas (ABG) analysis of an 8 week-old calf with bladder rupture performed 5, 120 and 180 min after starting the adrenaline infusion, ABG 1, ABG 2 and ABG 3 respectively. Reference intervals were taken from Dillane et al. [24]

Variables	Reference interval	ABG 1	ABG 2	ABG 3
**pH**	7.37–7.47	7.39	7.30	7.18
**PaO**_**2**_**(kPa)**	13.33–66.67	48.60	35.69	35.88
**PaCO**_**2**_**(kPa)**	5.77–7.82	5.63	6.09	6.76
**Bicarbonate (mmol/L)**	28.0–36.9	25.0	21.9	18.6
**Base excess (mmol/L)**	+ 2.6 to + 10.8	+ 0.1	−4.2	−9.1
**Lactate (mmol/L)**	0.42–0.75	2.76	5.69	8.78
**Sodium (mmol/L)**	133.3–140.2	131.7	134.1	134.1
**Potassium (mmol/L)**	4.13–5.41	6.90	5.08	4.63
**Ionized calcium (mmol/L)**	1.17–1.37	1.05	1.12	1.2
**Chloride (mmol/L)**	93–101	99	99	101
**Glucose (mmol/L)**	3.9–8.4	3.5	6.6	5.6

Partial pressure of oxygen in arterial blood (PaO_2_), partial pressure of carbon dioxide in arterial blood (PaCO_2_)

During surgery, 1600 mL of fluid was drained from the abdomen. A ruptured urachal abscess was found and removed and, the damaged bladder wall was resected and repaired. At the time of surgery the urethra was patent and no urethral strictures were evident.

Ketamine and adrenaline infusions were maintained until the end of the anaesthesia. The total duration of anaesthesia and surgery was 240 and 173 min respectively. At the end of anaesthesia the oral cavity was visualised with a laryngoscope and any regurgitant material was suctioned. The calf was then hoisted into a padded recovery box and placed in sternal recumbency with the head supported on a foam pad. Flow-by oxygen 8 L/min was administered during the recovery period and the animal was actively warmed. The calf was extubated and stood, 20 and 80 min after the end of anaesthesia respectively. During recovery, the calf was tachypnoeic (60 breaths per minute) with normal lung auscultation. Butorphanol (0.2 mg/kg) IV was administered to treat suspected pain and, RR decreased. Butorphanol (0.2 mg/kg) IV was administered every 2 h overnight. The calf received Hartmann’s solution 2.5–5 mL/kg/h during the hospitalisation period.

The day following surgery the calf was dull and presented with nasal discharge. Amoxicillin and clavulanic acid 1 mL/20 kg IM BID and meloxicam 0.15 mg/kg (Loxicom; Norbrook Laboratories Limited, Monaghan, Ireland) SID IV was commenced.

Two days following surgery, a perineal urethrostomy was carried out due to a suspected urethral obstruction. A sacrococcygeal epidural was performed with 0.1 mg/kg xylazine and 2 mg/kg lidocaine, followed by total intravenous anaesthesia with xylazine (0.01 mg/kg) and ketamine (1 mg/kg) IV boluses administered to effect. Flow-by oxygen, 8 L/min was administered and vital parameters (HR, RR) were assessed throughout the procedure. A venous blood gas performed after surgery showed normal lactate levels (1.17 mmol/L). Following the perineal urethrostomy the bladder was manually expressed every 4 h as the calf was not able to urinate, but the calf was brighter, standing for longer periods and able to eat.

One week after the first surgery, the calf was euthanised due to repeat urethral obstruction and severe abdominal distention. On post-mortem examination, severe fibrinous uroperitonitis, urethral fibrin cast and severe neutrophilic necrotising tubulonephritis were found.

## Discussion

This is the first case report that describes the administration of an adrenaline infusion as part of hypotension treatment in a cow and a calf during general anaesthesia. Hypotension is one of the most common complications that occurs during general anaesthesia [1, 2]. For the purpose of this discussion the definition of hypotension in bovines has been extrapolated from equine anaesthesia, and is defined as a MAP < 70 mmHg [5, 6, 8]. The cow and the calf in this case report had a MAP lower than 70 mmHg for at least 105 and 75 min respectively (i.e. from the onset of IBP measurement). This long duration of hypotension may have led to post-operative complications. In veterinary medicine, prolonged hypotension during equine anaesthesia has been associated with post-anaesthetic myopathy, neuropathy and organ injury, and may contribute to peri-anaesthetic deaths [5, 7]. No such evidence has been reported in bovines. It is possible subclinical organ injury occurred in these cases, but there was no evidence of post-anaesthetic myopathy or neuropathy. It is important to aggressively treat hypotension during anaesthesia. In this case report common techniques used to treat hypotension failed. Treatments included decreased delivery of inhaled anaesthetic agent, administration of anticholinergic drugs (i.e. atropine), increased intravenous fluid administration, use of MAC sparing drugs (e.g. ketamine infusion) and locoregional techniques (e.g. nerve blocks and epidural). The only treatment that appeared to be effective at increasing BP was the administration of adrenaline as an infusion.

Adrenaline has an immediate onset of action and a short duration (5–10 min) of action when administered as an IV bolus [25]. When administered as an infusion it can provide a sustained effect. Mink et al. [26] demonstrated that adrenaline infusions could effectively be used to treat cardiovascular collapse associated with anaphylactic shock during general anaesthesia. The rate of adrenaline required to maintain mean arterial pressure (MAP) at 70% of pre-shock levels was 0.19–0.45 μg/kg/min. There are no current recommendations for adrenaline infusion rates in bovines. However, it is possible to extrapolate the infusion rate of adrenaline used in dogs with that of bovines, if the relationship between metabolic rate and drug doses are taken into consideration. By using allometric scaling, the bovine dose when compared to the canine dose should be reduced by 90% to 0.02–0.04 μg/kg/min [27].

$${\displaystyle \begin{array}{c}\left( Bovine\ dose\right)\left( Bovine\ {mass}^{0.75}\right)=\left( Canine\ dose\right)\left( Canine\ {mass}^{0.75}\right)\\ {}\left( Bovine\ dose\right)\left( 480\ {kg}^{0.75}\right)=\left( 0.19\hbox{-} 0.45\mu g/ kg/\mathit{\min}\right)\left( 20\ {kg}^{0.75}\right)\\ {}\left( Bovine\ dose\right)(102.5)=\left( 0.19\hbox{-} 0.45\right)(9.5)\\ {} Bovine\ dose= 0.02\hbox{-} 0.04\mu g/ kg/\mathit{\min}\end{array}}$$

The most common side effects reported in the literature during adrenaline infusion include arrhythmias (ventricular tachycardia, bradyarrhythmias, ventricular premature complexes, ectopic beats and second-degree atrioventricular block) and hypertension. Arrhythmias have been induced in dogs with infusion rates ranging from 1.5–10 μg/kg/min [28–30], much higher infusion rates than those used in this case report. In case 1, the adrenaline infusion was started at 0.017 μg/kg/min, (a rate which had been extrapolated from dogs) and gradually increased to 0.05 μg/kg/min. This was associated with an increase in MAP and resolution of hypotension. However, it is possible that blood pressure increased because of nociception, as the effect of the block was waning, and repeat mandibular block failure. This is supported by the fact that blood pressure continued to increase despite the adrenaline infusion rate remaining constant and, later during weaning of the infusion. In case 2, the adrenaline infusion was started at 0.05 μg/kg/min and gradually increased to 0.25 μg/kg/min. Blood pressure increased in line with increases in the adrenaline infusion rate and MAP remained stable throughout the procedure.

The use of an adrenaline infusion in healthy human volunteers and cats is associated with increased HR, MAP and cardiac output [31, 32]. In this case report, HR did not significantly increase with the administration of adrenaline infusion rates ranging from 0.01–0.25 μg/kg/min and no arrhythmias were noted. Significant improvements in BP were only observed with infusion rates exceeding 0.05 μg/kg/min.

In case 1, the repeated mandibular block may have been unsuccessful due to the dose of the local anaesthetic given, or the technique used. In the cases discussed, lidocaine was used under the “cascade”, as the local anaesthetic to perform the mandibular block and the epidural. Lidocaine has the advantage of having a quick onset and intermediate duration of action (90–180 min) in ruminants [33]. The addition of adrenaline or an α-2 adrenergic agonist can slow the systemic absorption of local anaesthetics and could have been used in case 1 to increase the duration of action of lidocaine [34, 35]. This may have removed the need for the right mandibular block to be repeated. The dose of lidocaine used to perform the regional block can also affect duration of action. The maximum safe dose of lidocaine in cattle is 10 mg/kg [33]. Previous studies have shown that higher local anaesthetic doses and concentrations administered when performing locoregional blocks result in longer duration of sensory and motor blockade [36]. In this case, the dose of lidocaine used per animal was 2 mg/kg. Use of higher doses may have been associated with a longer duration of action removing the need to repeat the right mandibular block, however it was convenient to use the total volume of a 20 mL vial of lidocaine per site. Another explanation for the repeat block failure was the technique used. The mandibular blocks were performed blind (i.e. ultrasound guidance and peripheral nerve simulation were not utilised to aid needle placement). Needle placement was guided by anatomical landmarks [37]. In this case, the anatomical landmarks were difficult to identify due to soft tissue swelling associated with the fracture. The success rate of blind blocks has been reported to be low due to an inaccurate local anaesthetic administration, even with the use of larger local anaesthetic volumes [38].

In case 2, the epidural performed with lidocaine prior to premedication may have contributed to the hypotension seen during this case. Local anaesthetics administered into the epidural space are associated with hypotension due to sympathetic blockade resulting in vascular vasodilation [39]. Hypotension may also have been due to systemic vasodilation, as the calf was suspected of being septic. In this case an adrenaline infusion was deemed an appropriate treatment for vasodilation induced hypotension and therefore it was started promptly.

Increases in lactate concentration were observed in both cases with adrenaline infusion. An increase in lactate concentration and a decrease in pH have been associated with adrenaline infusions in the human literature, in both septic and healthy patients. A review, by Levy et al. [31], concluded that the increase in lactate observed with adrenaline is likely associated with increases in carbohydrate metabolism and not with cellular hypoxia. Pascoe et al. [32] reported a higher increase in lactate concentration in healthy cats after adrenaline infusion, compared with dopamine or dobutamine. The calf in this paper was suspected of having septic shock given a confirmed source of infection (septic umbilicus), and marked hypotension during anaesthesia. Septic shock is defined, as a persistent hypotension non-responsive to fluid resuscitation and requiring vasopressors to maintain MAP > 65 mmHg with serum lactate level > 2 mmol/L [40]. The increases in lactate seen during this case (2.76–8.78 mmol/L) may have been due to progression of septic shock and not solely due to the increasing adrenaline administration [41]. Another reason for the increasing lactate concentration may have been a decrease in lactate clearance, that can occur in animals with septic shock, rather than any increase in production [42]. With the removal of the focus of infection (septic umbilicus) the lactate decreased from 8.78 to 1.2 mmol/L in the 2 days following surgery. In case 1, only mild increases (0.43 to 1.2 mmol/L) in lactate levels were observed with the administration of an adrenaline infusion, with lactate levels remaining within the normal reference ranges. Only one arterial blood gas sample was assessed during anaesthesia in case 1, therefore further unobserved changes in lactate levels may have occurred.

It is possible that other steps could have been taken to manage hypotension in this case report including, administration of different doses and types of crystalloid fluids, higher doses of atropine, the use of α-2 adrenergic agonists as MAC sparing drugs or calcium gluconate as a positive inotrope.

Case 1 received Hartmann’s at 5–10 mL/kg/hr. with a total volume of 16 L (35 mL/kg) during the 230 min anaesthetic. The patient was estimated to be 6% dehydrated prior to anaesthesia indicating a 29 L fluid deficit. By the end of the anaesthetic 73% of the fluid deficit (21 L including 5 L IV fluids prior to induction) was replaced. It is standard practice within veterinary medicine in cases of dehydration that a third of the fluid deficit is replaced rapidly (2–3 h) and the remainder is replaced more slowly over 24 h [43]. In this case the rate of fluid resuscitation was faster owing to the severe hypotension during anaesthesia. Further fluid administration during anaesthesia may have been warranted, but effective fluid bolusing was challenging given the large size of the patient and the large volume of fluid required, which was in itself economically prohibitive.

Case 2 received Hartmann’s at 10 mL/kg/hr and a 20 mL/kg bolus of fluids with a total volume of 7 L (46 mL/kg) during the 240 min anaesthetic. Fluid administration in septic patients is usually based on the individuals fluid responsiveness. In this case changes in BP were used to assess fluid responsiveness. Despite a 20 mL/kg Hartmann’s fluid bolus, no increase in BP was observed. It is possible that a larger fluid bolus may have resulted in an improvement in BP, but further fluid administration may equally have resulted in oedema and organ dysfunction [44]. Cardiac output may have been a better indicator of fluid responsiveness [45] and may have altered the approach to fluid administration, but was unavailable in this case.

Other fluid options for the treatment of hypotension in these cases included hypertonic saline, which is licenced in food producing animals in Ireland, and has been shown when administered IV to have a positive impact on BP [8] but was not used in either of these cases. In case 1 the patient was expected to have third space (interstitial) fluid deficits. Intravenously administered hypertonic saline works by drawing fluid from the interstitial space into the intravascular space. Interstitial dehydration has been cited as a contraindication for the administration of hypertonic saline [46]. In adult bovines, it is recommended that 20 L of fluids should be administered IV or by orogastric tube after IV hypertonic saline administration [19]. In case 1, the concerns regarding damage to the surgical site precluded orogastric tube administration of fluids post-operatively. Hypertonic saline may have been an option for management of hypotension in the second case, given the severity of the hypotension and the smaller patient size, allowing the use of IV fluids in the immediate post-anaesthetic period.

Unfortunately colloids although likely to have been useful in both cases are not licenced in food producing animals and cannot be used.

In both cases atropine was used in an attempt to treat bradycardia. The expected HR for an adult bovine under general anaesthesia is 60–90 bpm [19]. Heart rate for 2 month old calves under general anaesthesia has not been reported, however the average HR for a 55–60 days old awake calf is reported to be 98 bpm [47]. In case 1, the dose of atropine used was 5 μg/kg IV, and this dose was repeated 10 min later, however the patient remained bradycardic (HR 40 bpm). In case 2, atropine 10 μg/kg IV was administered and repeated 10 min later, again the patient remained bradycardic (HR 55 bpm). The recommended doses for atropine reported in ruminants is 6–10 μg/kg IV and 20–40 μg/kg IM [19]. Side effects associated with the administration of atropine include decreased gut motility, viscous secretions (risk of airway obstruction) and atrioventricular blocks at low doses [48]. It is possible if larger doses of atropine had been used in both cases improvements in HR would have been observed, with a resultant improvement in BP.

The lack of licenced drugs in food producing animals makes maximisation of MAC sparing (decrease in the requirement of inhalant agent) through the use of regional anaesthesia an important part of food producing animal anaesthesia. Alpha-2 adrenergic agonist infusions are commonly used to decrease isoflurane requirements in horses [49]. In the cases discussed here, α-2 adrenergic agonists were not used due to a concern that further decreases in HR would be observed.

Calcium gluconate may have been an option to treat hypotension in case 1. Calcium gluconate works by increasing heart contractility [19], and the cow was hypocalcaemic possibly owing to her recent calving [50].

Other drugs commonly used in non-food producing animals for the management of hypotension include positive inotropes dopamine, dobutamine and nor-adrenaline however they are not licenced in food producing animals.

## Conclusion

Maintenance of normotension is important during bovine anaesthesia to prevent the development of post-anaesthetic complications including myopathy, neuropathy and organ injury. This case report describes the novel use of an adrenaline infusion to effectively treat hypotension during anaesthesia in 2 bovines when other conventional treatments failed. The only side effects noted with adrenaline infusion in this case report was an increase in lactate levels, however cardiac arrythmias and hypertension are also possible side effects. In practice, adrenaline infusion may present an option for the treatment of anaesthetic induced hypotension in bovines when other conventional treatments fail. Adrenaline infusions should be started at conservative rates and gradually increased, while continually monitoring ECG and BP. If arrythmias occur infusions should be reduced or stopped, and changes in blood lactate levels should be monitored throughout the adrenaline infusion administration.

Since writing this case report, the authors have used adrenaline infusions in one calf and two goats. The authors now routinely start adrenaline infusions at 0.05 μg/kg/min. In the most recent caprine case, the adrenaline infusion was increased to 2 μg/kg/min. A positive blood pressure response to adrenaline infusion administration has been seen on each occasion without any cardiovascular side effects. However, further research is required to establish the recommended infusion rates, cardiovascular effects and possible side effects of adrenaline infusion administration as a treatment for hypotension in bovines.

## Data Availability

The data used are available from the corresponding author on reasonable request.

## Abbreviations

MAP: Mean arterial pressure
MAC: Minimum alveolar concentration
IV: Intravenous
UCDVH: University College Dublin Veterinary Hospital
CRT: Capillary refill time
HR: Heart rate
bpm: Beats per minute
KCl: Potassium chloride
IM: Intramuscular
RR: Respiratory rate
ETiso: End-tidal isoflurane
ETCO_2_: End-tidal cardon dioxide
TV: Tidal volume
PIP: Peak inspiratory pressure
SpO_2_: Oxygen saturation
ECG: Electrocardiogram
IBP: Invasive blood pressure
BP: Blood pressure
SID: Once daily
BID: Twice a day
ID: Internal diameter
